# Mobile eye tracking applied as a tool for customer experience research in a crowded train station

**DOI:** 10.16910/jemr.16.1.1

**Published:** 2023-01-16

**Authors:** Andrea Schneider, Beat Vollenwyder, Eva Krueger, Céline Mühlethaler, Dave B. Miller, Jasmin Thurau, Achim Elfering

**Affiliations:** University of Bern, Bern, Switzerland; Ecole Polytechnique Fédéral de Lausanne EPFL, Lausanne, Switzerland; Swiss Federal Railways SBB CFF FFS, Switzerland; Tufts University, USA

**Keywords:** Mobile Eye Tracking, Crowding, Wayfinding, Train Station, Customer Insights, Crowd Density

## Abstract

Train stations have increasingly become crowded, necessitating stringent requirements in the design of stations and commuter navigation through these stations. In this study, we explored the use of mobile eye tracking in combination with observation and a survey to gain knowledge on customer experience in a crowded train station. We investigated the utilization of mobile eye tracking in ascertaining customers’ perception of the train station environment and analyzed the effect of a signalization prototype (visual pedestrian flow cues), which was intended for regulating pedestrian flow in a crowded underground passage. Gaze behavior, estimated crowd density, and comfort levels (an individual’s comfort level in a certain situation), were measured before and after the implementation of the prototype. The results revealed that the prototype was visible in conditions of low crowd density. However, in conditions of high crowd density, the prototype was less visible, and the path choice was influenced by other commuters. Hence, herd behavior appeared to have a stronger effect than the implemented signalization prototype in conditions of high crowd density. Thus, mobile eye tracking in combination with observation and the survey successfully aided in understanding customers’ perception of the train station environment on a qualitative level and supported the evaluation of the signalization prototype the crowded underground passage. However, the analysis process was laborious, which could be an obstacle for its practical use in gaining customer insights.

## Introduction

During rush hours, thousands of commuters rush through highly frequented train stations either towards the exit or train platforms. This phenomenon is interesting from two perspectives: (1) train station management, which faces the challenge of optimizing train stations capacities to ensure safe operation and good customer experience and (2) commuters who need to navigate through a crowded environment. However, investigating commuter/customer perception poses methodological challenges. In this study, we explored the use of mobile eye tracking to gain customer insights or knowledge about the customer (60) in crowded train station environments and to learn more about path choices in crowded field settings. This study was conducted at a train station in Switzerland in collaboration with the SBB (Schweizerische Bundesbahnen, Swiss Federal Railways). The focus was on a crowded passage, a bottleneck, which leads to the platforms where up to 2,000 commuters walk through during rush hours.

### Crowding and wayfinding

Previous studies have mainly reported on the negative effects of crowding (an aggregation of people in the same space at the same time) (1), including its effects on humans in rail contexts (53). “Crowding” is frequently used to describe psychological tensions experienced in crowded areas. Evans and Wener (15) describe crowding as a state that occurs when the regulation of social interaction is unsuccessful, and the desired level of social interaction is exceeded by the actual amount of social interaction experienced. Hence, crowding is often associated with negative impacts on experience, including psychological or physical discomfort, perceptions of risk to personal safety or security or actual risks to safety (10; 11; 53).

Previous studies have reported that the objective reality of crowd density, (a numerical measure of the concentration of individuals within a given geographical unit) need not be congruent with its subjective perception (48). A distinctive comparison between the perception and objective reality of crowd density, indicates that there is a subjective perception of safety and an objective measurable safety (61). Objective safety refers to the actual number of accidents or incidents, while subjective safety perception describes the feeling of perception of safety or people’s subjective experience of accident risk. Therefore, the subjective experience of commuters who have to find their way through the crowd either towards the exit of the train station or platform may have differences because the crowd density might be individually perceived, depending for example on cultural background (19).

In architectural design, wayfinding refers to the user experience of orientation and choosing a path within a built environment (29). Lynch (38) defined wayfinding as the “consistent use and organization of definite sensory cues from the external environment.” Environmental psychologists subsequently extended the definition to include the use of signage and other graphical and visual clues that aid orientation and navigation in built environments (2).

Previous studies have explored the collective patterns of pedestrian movements, such as the formation of pedestrian lanes and circulating flows at intersections, using empirical observations (41, 42), video-based experiments wherein the motions of pedestrian groups were recorded by video (43; 69), and simulation studies (23, 22; 45). These studies have demonstrated that various factors, including environmental layouts (e.g., entrances, exits, walls, and obstacles) and interactions with other pedestrians, can influence an individual's locomotion. For example, Yi et al. (69) found that stationary crowds can dramatically decrease walking efficiency in terms of walking distance and travel time; however, moving crowds (even at high densities) did not affect pedestrian flow. Studies on the effects of crowds on human navigation, especially in real-life contexts, have reported that herd behavior influences wayfinding. Van den Berg et al. 2018 define herd behavior as the phenomenon of individuals seeing other people doing something and believing that what they are doing is a good alternative, and imitating their actions. Several researchers have reported that stressed-pedestrians follow other pedestrians (23; 30; 31; 42). This pattern was found during a real-life evacuation (i.e., collective herding effect; 23) and during experiments in both real and virtual environments (30; 31; 42). Conversely, Bode et al. (4) reported that the pedestrians are influenced by other pedestrians and other sources of directional information, such as signs and previous experiences as well. Participants did not tend to follow the crowd unless the sign and crowd provided conflicting information.

A study on the effect of crowdedness on human wayfinding and locomotion in a multi-level virtual shopping mall by Li et al. (36) reported that crowdedness did not affect wayfinding strategies or initial route choices; however, it did affect locomotion of participants who were more likely to avoid crowds by moving close to the boundaries of the environment in conditions of high crowdedness. Moreover, the structure of the virtual building appeared to affect the wayfinding strategy. They concluded that both physical and social environments influence wayfinding in multi-level buildings.

To summarize, normative influences including herd behavior, signs, physical environments, and previous experiences apparently influence wayfinding. Context and specific situations are important aspects in determining the decisive influencing force. Therefore, the influence of other pedestrians on path choice, and the influence of signaling on pedestrians in a moving crowd need further investigation. The literature on the psychological effects of crowding suggests not only that other pedestrians influence path choice but also customer experience.

### Customer experience and understanding the customer perspective

Providing a good customer experience is an important management objective (35) that is crucial for achieving profitability (9; 24). Customer experience is associated with all direct and indirect interactions that a (potential) customer has with a company, its services, products, or brand (7). The increasing possibilities to share experiences via mobile apps, review websites, or social media, have made customers increasingly empowered to choose their products or services in a competitive marketplace (62); thus, they have higher expectations regarding products and services (47). Hence, a better understanding of customers and creating a good customer experience has become imperative in recent years (58).

Customer experience management aims towards a better understanding of customer experience wherever a customer-company interaction occurs; therefore, different methods to generate customer insights are applied. This is a part of the applied user- or human-centered approach (e.g., 5), which has been pursued by the SBB in various areas (e.g., train station platform design (55; 53), websites, in-house applications (e.g., 56; 66), and accessible mobile applications (65). Current discussions on methods to gather customer insights include suggestions to collect retrospective data or data from a subjective perspective (51) in real-time and using newer innovative methods (55; 53). Furthermore, Said et al. (51) contend that while there is significant literature on the necessity and influence of customer insights on decision-making, there is a lack of literature exploring the generation of customer insights in real environments. Collecting customer insights and investigating of customer experience from a holistic perspective in train station environments are yet to be thoroughly investigated. Therefore, mobile eye tracking may be an innovative method to better understand customer experience at train stations.

### Mobile eye tracking in the field

Eye tracking is an established method for research on visual attention. Mobile eye tracking facilitates examination of gaze behavior in real-world pathfinding tasks. It allows eye tracking research to be conducted in real-world environments using head-worn systems due to its mobility and context- relatedness. Ambulatory head-mounted eye tracking obtains both eye movement parameters and point- of-view video recordings. Thus, participants' eye fixation and movement can be superimposed on the video recordings of their views. This enables capturing of the surrounding real-world environments (40). Mobile eye tracking has been used in different areas, including marketing (e.g., 33), tourism (for a review see 57), usability (e.g., 44) and safety (40). Martinez-Marquez et al. (40) reported that mobile eye tracking is widely used to research the visual, cognitive, and attentional aspects of human mental performance in applied safety areas, including aviation (e.g., 32; 50), maritime (e.g., 20), construction (e.g., 21) and transportation (e.g., 17). In these studies, eye tracking helped to improve human- machine interaction and was applied for design improvement or situation-awareness training.

Furthermore, previous studies have reported the usefulness of eye tracking in train stations (8; 68). Mobile eye tracking was explored in a methodological triangulation (observation of path choice behavior, measuring gaze behavior with eye tracking, and assessing confidence ratings through interviews) by Buechner et al. (8) to compare two design solutions. Buechner et al. (8) proposed that, rather than general guidelines, a methodology for empirically comparing design solutions is required. The methodological combination resulted in an improved situational analysis than when only one method was used. The combination of a forced choice paradigm, questionnaires, and eye tracking is based on cognitive and environmental psychology to combine visual attention data with observations of behavioral decision-making and self-assessment. In a real-world wayfinding task at a train station, eye tracking was successfully used for the design and placement of signs (68).

The analysis of real-world eye tracking data remains challenging. The definition of fixations and gaze behavior differs between studies conducted in laboratories or controlled settings (e.g., driving (39) or marine operation simulators (18)) and studies conducted in natural environments (e.g., outdoor (14)). The use of areas of interest (AoI) is a procedure typically applied in laboratories or controlled settings. Babu et al. (3) used Areas of Interests (AoI) to analyze saccadic gaze movements between and within these areas of interest. Wolf et al. (67) developed an algorithm that automatically maps gaze data onto respective AoIs. However, Evans et al. (14) addressed the challenges in collecting and analyzing eye tracking data in outdoor environments. The environment is dynamic, and the gaze data are recorded relative to the user’s perspective (without a link to absolute coordinates); hence, gaze tracking data often need manual classification after a frame- by-frame review of video data of the user’s gaze to determine where the user’s point-of-view. Kredel et al. (33) addressed the challenge of analyzing natural gaze behavior and concluded that studies collecting gaze behavior in natural settings have used manual analysis, even though it may not be accurate, due to the unavailability of better solutions.

Studies using eye tracking in laboratories or controlled settings usually use established algorithms or techniques to identify fixations (e.g., 52; 3; 67). Salvucci and Goldberg (52) addressed the process of fixation identification (i.e., separating and labeling fixations and saccades in eye-tracking protocols) and proposed five algorithms. Fixations can be identified based on either spatial (velocity-based, dispersion-based, or area-based) or temporal (duration-sensitive or locally adaptive) criteria. However, in addition to this computational definition (i.e., representation of an event in the eye tracker signal), which is often used in laboratories, there is a functional definition of fixations (i.e., purpose of the eye movement) (25). In functional definition, fixations can be analyzed in terms of either spatial (i.e., where the participants are looking), numerical (how often someone is looking in a certain area), or personal factors (i.e., is there a difference between diverse groups of people, e.g., depending on cultural background (59)) (26). Reitstätter et al. (49) used a functional definition in their study on the perception of art in a museum and used a manual annotation process for eye-tracking videos. Jovancevic-Misic and Hayhoe (28) treated a “fixation” as the period when gaze remained at the same location with respect to the pedestrian, although it included some smooth rotational component and is specified as “gaze”. Steil et al. (63) stated that they used the term fixation to jointly refer to users’ visual focus of attention on a gaze targets irrespective of scene and head motion. However, looking or gazing at a stimulus or object does not necessarily indicate that attention is paid towards that said stimulus or object (i.e., “inattentional blindness” (e.g., 27)). The detection of visual attention is remains under research (34; 54).

To summarize, mobile eye tracking facilitates the collection of gaze behavior in natural settings, although the data analysis remains challenging. Studies conducted in laboratories can be analyzed by algorithms, which leads to a high level of accuracy, while studies in natural settings require manual analysis.

### Present study

In this study, we explored the potential of mobile eye tracking as a tool for research on customer experience and investigated the feasibility of eye tracking for customer experience management. We attempted to analyze attention to visual pedestrian flow cues in a train station using mobile eye tracking. Based on the existing literature, we hypothesized that visual cues will be considered when crowd density was low and that normative influences such as herd behavior will be strong when crowd density was high.

## Methods

### Participants and Design

A total of 19 participants (age *M* = 29, *SD* = 5, range 18–51 years) completed the eye tracking study. Participants were recruited from a circle of acquaintances of the study coordinators and were familiar with the train station. Participants participated out of their interest in innovative methods and received a local sweet treat as compensation. A between-study design with control and post-intervention conditions was applied. Seven participants were assigned to the control condition and nine to the post- intervention condition. The eye-tracking data of 3 participants were excluded because of insufficient tracking quality.

### Materials

#### Subway and Signaling

**Figure 1.**
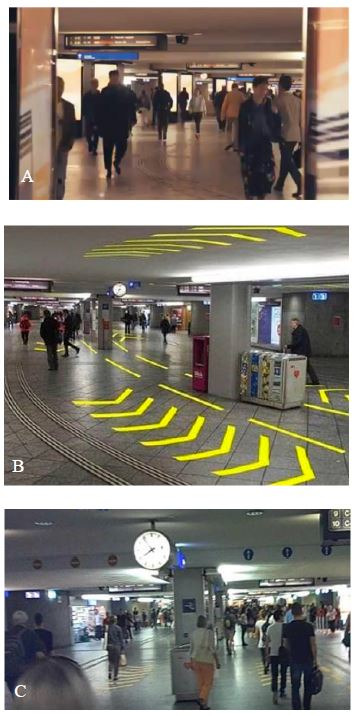
Train station passage before implementation of the signaling prototype (control condition) (A). The yellow arrows on the floor and ceiling, and the blue and red one-way signals at the entrance of the passage are installed as a prototype to regulate pedestrian flow (post-intervention condition) (B and C).

The investigated location was an underground passage at the main train station in Bern (Switzerland); this is a nodal train station in Switzerland with high traffic. It can be entered via the train station platform side, or via the two main exits or entrances (see Figure 1). One exit/entrance leads to the bus/tram station or storage area, and the other to the elevators, which leads to the university campus above the train station. Commuters can buy food and beverages from both sides of the underground passage. In particular, during rush hours, the passage is crowded.

In the post-intervention condition, yellow arrows, to indicate the required walking direction, were installed. These arrows were fixed to the ceiling and floor. Therefore, the two lanes in the middle of the passage would function as fast lanes, while the space closer to the walls could be used normally. One- way signs were installed at the entrance of the passage (see Figure 1). These signals are commonly used as signs of road traffic in Switzerland, and were familiar to the population. The signal setup was implemented as a prototype to explore the effects of the signs to regulate pedestrian flows.

#### Mobile Eye Tracker

Eye movements were recorded using SMI eye-tracking glasses (SMI ETG 2w). Mobile eye- tracking glasses facilitate the measurement of real-time natural gaze behavior. The system has a sampling rate of 120 Hz and tracking accuracy of approximately 0.5° of visual angle. The glasses were connected to an SMI ETG 2w smart recorder based on Samsung Galaxy Note 4. The smart recorder was placed in a jacket or trouser pocket and permitted free movement during the experiment. Before the experiment, three-point calibration was performed. The data were converted into gaze mapping videos using BeGaze 2 software from SMI.

### Procedure

The participants were brought to a specific starting point and provided instructions on their destination. They were asked to imagine a familiar situation to make the situation more realistic (scenarios were created; Table 1 and Table 3 in appendix). The order of the scenarios was randomized. During the scenarios, the participants' eye movements were recorded using the eye-tracking glasses. The eye-tracking glasses were set up and calibrated either in the train while traveling to Bern or outside the train station for participants who started from the bus station. After the eye-tracking measurement, participants were asked to rate their comfort levels in the underground passage, their perception of the crowd’s density, and if they noticed anything unusual.

#### Scenarios

**Figure 2.**
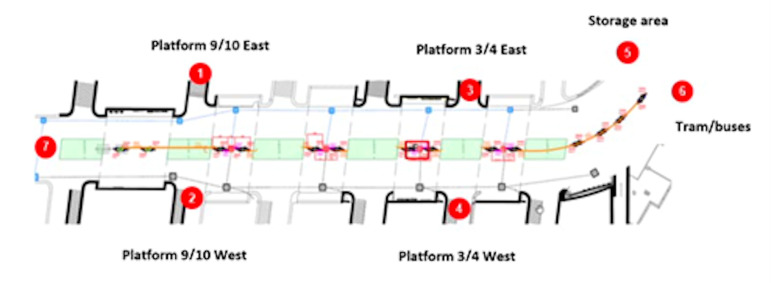
The investigated passage leads to the train station platforms. Access to the platforms is possible by taking a stair or ramp on either the left- or right-hand side of the passage. The red points mark the various starting points and aims for the scenarios. Additionally, points 7 and 6 mark the main entrance/exit of the train station.

Scenarios were created to help participants immerse themselves in familiar situations. Different scenarios were created because generally commuters enter and leave the underground passage from different starting and ending points. The starting and ending points are illustrated in Figure 2, and the scenarios are elucidated in Tables 1 and 3 (appendix). We focused on the movements when walking through the underground passage and on the influence of the implemented signs on the movements.

### Measures

Gaze behavior was recorded using eye tracking glasses. Points of interest were predefined as the analysis protocol for gaze behavior. In the control condition (no implemented steering signs), the following points of interest, which are important customer touch points in the passage, were determined: (1) shopping information; (2) pictogram with platform number; (3) big departure monitor and information sign; (4) small departure monitor; (5) transition map; and (6) other passengers, (7) locker pictogram, (8) general information signs. In the post-intervention condition (with the implemented visual cues), the following points of interest were added: (9) direction sign (arrows) on the floor, (10) direction sign (arrows) on the ceiling, and (11) one-way direction sign. Furthermore, the perception of a point of interest led to a change in behavior (walking direction or walking velocity) was analyzed.

**Figure 3.**
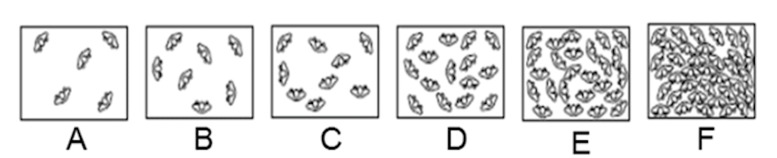
The LOS scale developed by Fruin and Benz (16) contains 6 pictures illustrating 6 different density levels, starting with the least crowded (A) and increasing linearly to the last and most crowded (F). (Figure from Fruin and Benz (16)).

The comfort level was assessed in an interview after the scenario on a 6-point scale (very comfortable, comfortable, rather comfortable, rather uncomfortable, and very uncomfortable). Estimated crowd density was measured using the level of service (LOS) scale developed by Fruin and Benz (16) (see Figure 3).

- LOS A: Standing and free circulation through the queuing area is possible without disturbing the others within the queue. The average pedestrian space (APS) was >1.2 m2/p.
- LOS B: Standing and partially restricted circulation to avoid disturbing the others in the queue is possible. (APS >0.9–1.2 m2/p).
- LOS C: Standing and restricted circulation through the queuing area by disturbing the others in the queue is possible. This density was within the range of personal comfort (APS >0.6–0.9 m2/p).
- LOS D: Standing without contact is possible. Circulation is severely restricted within the queue, and forward movement is only possible as a group. The long-term waiting at this density is uncomfortable. (APS >0.3–0.6 m2/p).
- LOS E: Standing in physical contact with others is unavoidable. Circulation in the queue is impossible. Queuing can only be sustained for a short period without serious discomfort. (APS >0.2–0.3 m2/p). LOS F: Virtually all persons in a queue stand in direct physical contact with others. This density is extremely uncomfortable. No movement is possible in the queue. There is potential for panic in large crowds at this density. (APS <0.2 m2/p).

**Table 1.** A selection of the scenarios containing the tasks for the participants are described. Each task had a starting and ending point. The numbers are illustrated in the map in Figure 3. A description of all scenarios is presented in the appendix (Table 3).

Scenario	Starting point	Aim	Scenario description
Catching a connecting bus	platform 9/10 east (1)	Tram/bus station (6)	“Imagine that you just arrived on platform 9/10. Now you must catch your bus in front of the train station.”
Finding a luggage locker	platform 9/10 east (1)	Storage area (5)	“Imagine that you just arrived on platform 9/10. Want to bring your luggage to the locker.”
Catching a connecting train on the same side of the passage	platform 9/10 east (1)	Platform 3⁄4 east (3)	“Imagine that you just arrived on platform 9/10. Now you must catch your connecting train on platform 3⁄4 east.”
Passing through the train station only.	Tram/bus station (6)	Train station exit toward the university (7)	“Imagine that you just arrived with the bus at the train station. Now you want to go to university.”

### Data Analysis

The eye tracking videos were analyzed using the BeGaze 2 software from SMI. Since we used a functional definition of fixation as judged by a human, similar to Reitstätter et al. (49), a manual annotation process was applied to define gazing behavior in a quantitative and descriptive manner. Therefore, we analyzed whether participants looked at predefined points of interest per scenario. Two analysts viewed at every video from start to end, including every test run separately, and individually determined the fixations. Subsequently, they consolidated their results on the determined number of fixations within every test run for interrater reliability.

Descriptive statistics were used to describe fixated points of interest (signs). A person- correlation was calculated for comfort level, estimated crowd density, and the number of gazes at visual cues (arrows on the floor and ceiling). A Mann-Whitney test was used to calculate the differences between the control and intervention conditions for comfort level and estimated crowd density.

## Results

### Gaze Behavior

#### Signalization

**Figure 4.**
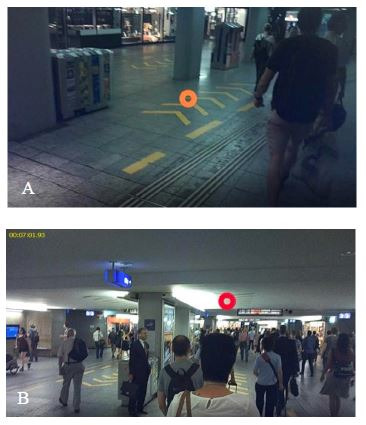
Approximately two thirds of the participants look at the signs on the floor (A), one third at the signs on the ceiling (B).

**Figure 5.**
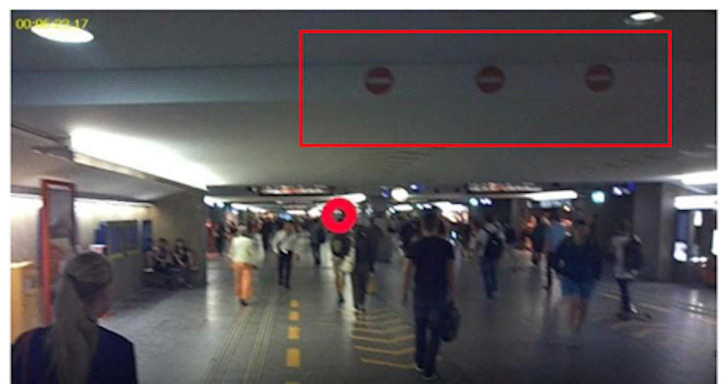
For most of the participants, the one-way street signaling on the ceiling is not in their visual field.

During the eye tracking session, 66% (6) of the participants fixated on the direction sign (yellow arrow) on the floor (Figure 4, left) and 33% (3) and on the ceiling (Figure 4, right), respectively, while 11% (1) looked at the direction sign at the entrance of the passage. For the rest of the participants, the direction signs at the entrance of the passage were not in their visual field (Figure 5).

When entering the underground passage, 56% (5) of participants used other pedestrians as orientations when choosing a path through the passage, while 45% (4) looked at the signs for orientation. Thus, a positive correlation between looking at other pedestrians as orientation when choosing a path at entrance and estimated crowd density (r (8) = 867, p < 0.05) was observed.

#### Standard way finding signalization and other pedestrians

**Figure 6.**
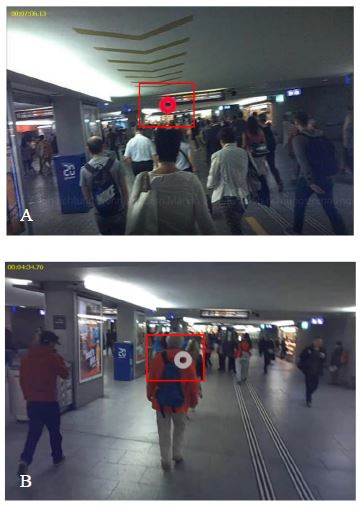
The departure information at the ceiling is fixated on by a majority of participants (A). Qualitative analyses of eye tracking data reveals that in the control and post intervention conditions overall attention is directed towards other pedestrians who are in front of the test participants (B).

**Figure 7.**
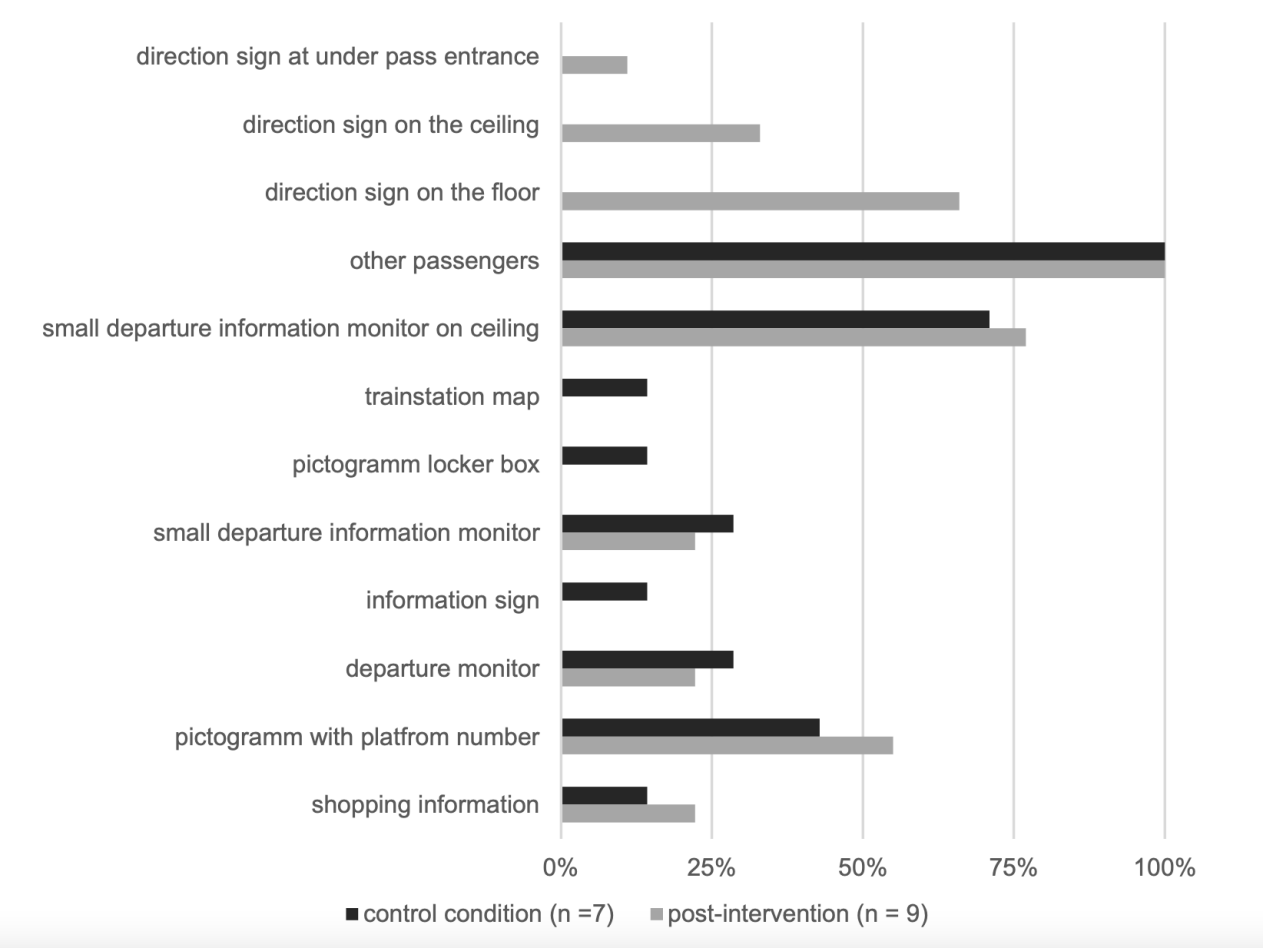
A summary of the gazed at points of interest in the control (pre-intervention) and post intervention conditions.

During the scenario, participants looked, on an average, at 4 points of interests (SD = 2, range = 2–7). A total of 71% (5) of the participants were looking at the small departure information monitor suspended from the ceiling, 43% (3) at the pictogram with the platform number, 29% (2) at the main departure monitor, 29% (2) at the small departure monitor, and 14% (1) at shopping information, the pictogram “locker box,” and transition map (Figure 6, left). All participants gazed repeatedly at other pedestrians in front of them (Figure 6, right). The summary of the gazed at points of interest are presented in Figure 7.

### Subjective measures

#### Perceived comfort

The perceived comfort in the control condition (M = 4.28, SD = 1.12) did not significantly differ from the post-intervention condition (M = 4.21, SD = 0.83, U = -.056, p=0.9). The points of criticism in the control condition were the narrow architecture (4), crossing people (1), lacking signalization (guidance to buses) (1), and the people clustering at certain locations in the underground passage (e.g., in front of the ticket machine) (1). In addition, participants who did not feel comfortable stated that the train station atmosphere was stressful due to crowding. The points of criticism in the post- intervention condition were similar to those in the control condition. Narrow architecture 44% (4), crossing people 33% (3), lacking signalization 11% (1), and people clustering at certain locations in the underground passage 11% (1).

#### Estimated crowd density

The estimated crowd density in the control condition (M = 3.28, SD = 1.25) did not significantly differ from the perceived estimated crowd density in the post intervention condition (M = 3.77, SD = 0.97, U = -.831, p = 0.39).

#### Correlations between estimated crowd density, comfort, and perception of direction signs (arrows)

Perceived comfort and estimated crowd density was negatively correlated (r (16) = -0.85, p<0.01). Participants were more comfortable when the estimated crowd density was lower and less comfortable when the estimated crowd density was high.

Perceived estimated crowd density and observing an arrow on the floor were negatively correlated (r (9) = -0.68, p = 0.04). Therefore, a higher the estimated crowd density indicated that the arrows on the floor were observed less. Perceived comfort and observing arrows on the floor were positively correlated (r (9) = .8, p = 0.01). Therefore, participants who were less comfortable were least likely to observe the arrows on the floor.

**Table 2.** Summary of Correlations, Means and Standard Deviations for estimated crowd density, comfort, and number of gazes at arrows on the ceiling and floor, respectively.

	*M* (*SD*)	Estimated crowd density	Comfort	Perception of arrows on ceiling	Perception of arrows on floor
Estimated crowd density	3.78 (0.97)	-	-.85**	-.34	-.68*
Comfort	4.22 (0.83)	-.85**	-	.79	.8*
Perception of arrows on ceiling	1.33(0.5)	-.34	.79	-	-
Perception of arrows on floor	1.67(0.5)	-.68*	.8*	-	-

Note. n = 9. * = p<0.05, ** = p<0.005

**Figure 8.**
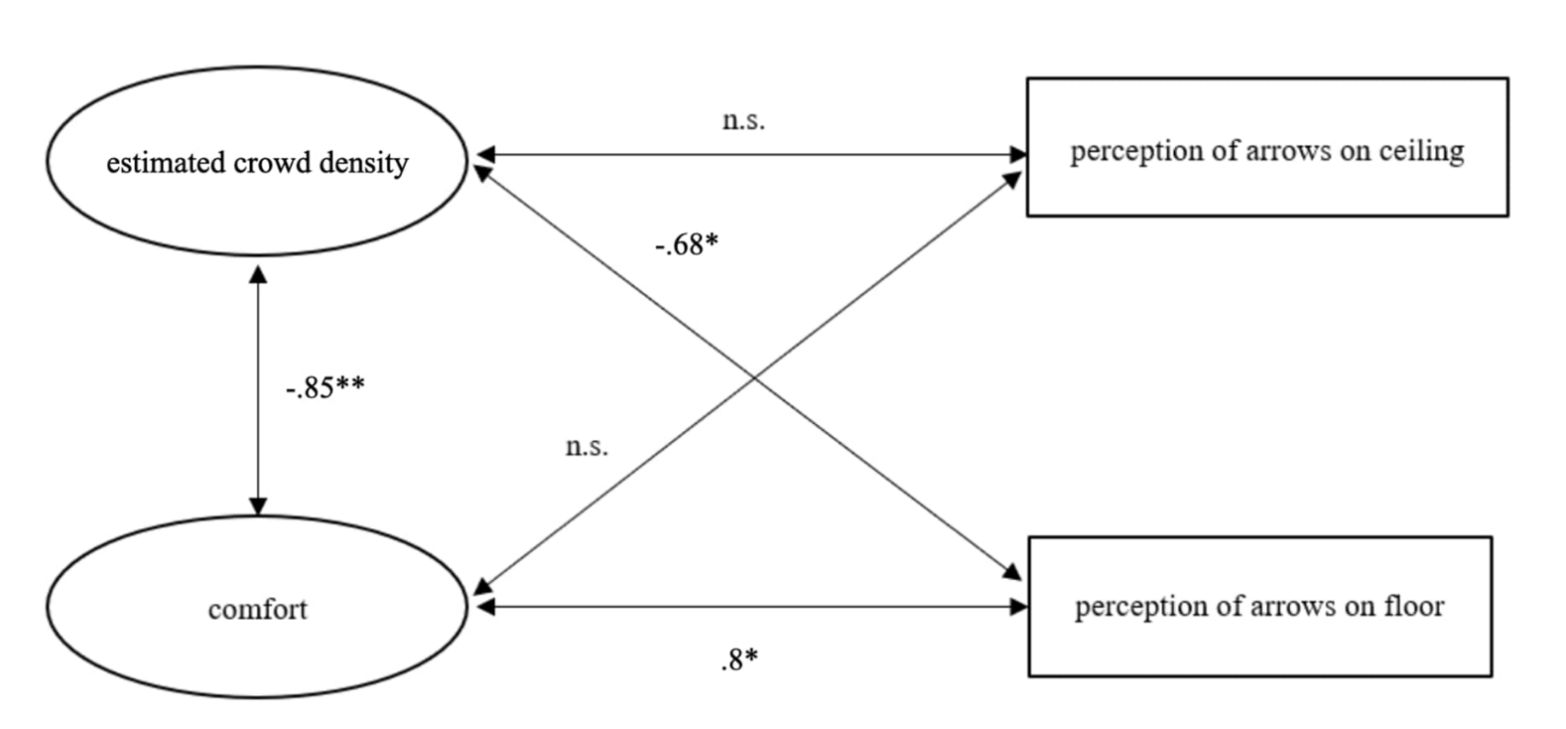
Illustration of correlations between estimated crowd density, comfort and number of gazes at arrows on the ceiling and floor, respectively.

## Discussion

In this study, we employed mobile eye tracking to analyze train station environments to gain customer insights and optimize this environment according to customer needs. We focused on the analyses of crowded train stations during rush hours and of the effect of visual flow cues on pedestrians’ path choice.

### Path choice during rush hour and the influence of implemented visual cues vs. other pedestrians

Our hypothesis that visual cues are more considered when the crowd density in the train station is low, and vice versa was confirmed. Our study indicated that the implemented signs were not effective when the crowd density was high. Nevertheless, other pedestrians influenced path choice behavior in the control (no signalization prototype) and post-intervention (signalization prototype implemented in the train station) conditions.

We concluded there are various reasons for the signs not being considered in the post- intervention condition. A possible reason may be because the signs were not necessarily in the field of view. Additionally, when the crowd density was high, the signs on the floor were unnoticed, possibly because they were not visible through the crowd. Conversely, we identified other pedestrians as influencing factors for path choice. Bode et al. (4) reported that pedestrians tend to “follow a crowd” in the absence of signs and that the crowd provide conflicting information. This is consistent with the finding in our study. Pedestrians chose to follow other pedestrians when entering the underground passage, especially in situations of high crowd density. Therefore, our results indicate that the observed passenger behavior was influenced by the behavior of others. Herd behavior has been observed in stressful situations (23; 30; 31; 42) such as evacuation (23); and therefore, this could additionally explain the behavior of the participants in our study.

### Experienced crowd density and comfort

Perceived comfort in the train station was rated high. According to the scale of Fruin and Benz (16) scores beyond a density of LOS C are perceived as uncomfortable. Although most participants chose LOS C or higher, they indicated feeling “comfortable” or “rather comfortable.” However, the indicated LOS correlated with the indicated comfort level as anticipated; a higher indicated LOS corresponded with a lower comfort level. These findings are consistent with those from previous studies, which found a relation between crowd density and perceived comfort (11; 53).

### Mobile eye tracking as a tool for customer experience research

Mobile eye tracking has proven to be a useful instrument for observing underground passages from the customers’ perspective. Observation or behavioral tracking tools can help understand an individual’s behavior in a train station environment. Interviews or surveys can help understand subjective perceptions or opinions. Eye-tracking adds another dimension to the survey of customers in train station environments. It helps understand customers’ visual perception of an environment. Therefore, our study is in line with previous literature that suggests exploring new methods to generate customer insights (51; 55, 53) and obtain a holistic picture of actual customer experience by adding an additional dimension to the exploration of pedestrians in train stations using methodological triangulation (8).

Moreover, the videos could be presented to the management to transport them to the customers’ perspective. This is valuable, as most of the gathered customer insights are presented as numbers or diagrams, which can be abstract and might not be representative of the actual customer (human) experience. Although it is implied that humans are the center of the customer experience concept, actual videos from the customer’s perspective in the respective environment or during a specific task can help empathize with the customer as a human being.

The exploration of customer insights in train stations may not classically be viewed or debated as a customer experience management issue, and is often left to the field of engineering. However, customer experience focuses on marketing or digital areas, and the train station is a relevant part of the customer journey; therefore, we contend that the methods employed in user- or human-centered design should similarly be considered for train stations. In our study, eye tracking helped understand why signalization did not work (on a behavioral level) as intended. We found that the signalization was neither seen nor was it the strongest factor determining path choice. These findings can provide crucial insights for future design decisions to improve pedestrian flow, which is relevant for a good customer experience and is a fundamental in ensuring safety in train station environments.

### Feasibility of mobile eye tracking for customer experience practitioners

Our study revealed that eye tracking research is challenging. The data collection was simple and convenient because of the ease of use of the smart recorder. The participants were able to behave naturally during the experiment and wearing the glasses in a public setting did not attract attention. However, the analysis and use of the data posed some challenges. First, the analysis of exported videos was time-consuming. The fixation points were manually determined. This is in contrast to the analysis of data collected in a controlled environment (e.g., a cockpit) or even on a computer screen (e.g., a usability test), where the coordinates within the field of recording can be determined and subsequently, used for an automated analysis using the respective software. Second, gaze fixation determination was difficult. Rapid gaze behavior is difficult to follow; therefore, it is exhausting and additionally, enhances the probability of misinterpreting the occurrence of fixations. Furthermore, differentiating a fixation from gaze gliding over an object is problematic, which leads to a discussion of whether “looking at something is seeing it” (34). This was especially difficult to determine because the participants were instructed to behave naturally; therefore, manual analysis is prone to error.

We recommend the use of mobile eye tracking to evaluate placements of pictograms, explore differences between customer segments (i.e., commuter, elderly people, and tourists), or research orientation in train stations. However, we have noticed that mobile eye tracking is associated with a certain amount of effort. Therefore, further technological advances in the use of automated coding in moving environments are required.

Newer models of mobile eye trackers claim to measure eye movements with a higher speed and accuracy, which could potentially measure smaller saccades or microsaccades and allow research in inattentional blindness, even in applied settings. This could prove noteworthy in evaluating safety experiences as proposed by Krueger, Schneider et al. (34) or Schneider et al. (53).

Eye tracking and eye movement research is rapidly advancing and trending. It presents ample opportunities in the field of human-machine interaction, customer or user experience in various areas. However, several ethical questions need to be considered. Although attention tracking during safety tasks may prevent the occurrence of accidents (e.g., 32; 50; 53) or improve design to support human beings to move safely or comfortably, the potential of misuse of such an instrument cannot be denied. The line between the agreement to be tracked or not could become blurry with future technological advances. For example, in our experiment the eye tracking instrument used clearly required consent because it required the participant to wear eye tracking glasses and carry a smart recorder; however, technologies like newer smartphone models, include eye tracking technology, which might be unknown to various users. Therefore, we propose that the use of an instrument that measures eye movements or even attention (e.g., through an integrated analysis of microsaccadic eye movements) should only be used in areas where value in terms of safety or comfort is created for the tracked human.

### Ethical aspects

Eye tracking and eye movement research is rapidly advancing and trending. It presents ample opportunities in the field of human-machine interaction, customer or user experience in various areas. However, several ethical questions need to be considered. Although attention tracking during safety tasks may prevent the occurrence of accidents (e.g., 32; 50; 53) or improve design to support human beings to move safely or comfortably, the potential of misuse of such an instrument cannot be denied. The line between the agreement to be tracked or not could become blurry with future technological advances. For example, in our experiment the eye tracking instrument used clearly required consent because it required the participant to wear eye tracking glasses and carry a smart recorder; however, technologies like newer smartphone models, include eye tracking technology, which might be unknown to various users. Therefore, we propose that the use of an instrument that measures eye movements or even attention (e.g., through an integrated analysis of microsaccadic eye movements) should only be used in areas where value in terms of safety or comfort is created for the tracked human.

Furthermore, eye trackers record physical environments and people in the vicinity are identifiable if their faces are visible. This is problematic as it is practically impossible to obtain consent from each person in the scene. Additionally, eye movements are potentially related to personal identification (13). Various methods were investigated to identify or manage privacy-sensitive image content. Orekondey, Schiele and Fritz (46) created a Visual Privacy Advisor Model that predicts user specific privacy score from images in order to enforce the users' privacy preferences. PrivacEye detects privacy-sensitive everyday situations and automatically enables or disables the eye tracker's first-person camera using a mechanical shutter (63). Bozkir et al. (6) proposed a novel transform-coding based differential privacy mechanism to further adapt it to the statistics of eye movement feature data and compared various low-complexity methods.

### Conclusion

Eye tracking is a useful tool for customer experience research which provides insights. Therefore, it helps explain certain behavior or customer experiences. In our case, herd behavior was identified as an influence for path choice in an underground passage. The presentation of eye tracking videos can help empathize with customers and adopt a customer perspective. Additionally, it provides specific indications on optimizing a certain design according to customer needs. However, the feasibility of the use of eye tracking for customer experience practitioners is limited. The analysis is time- consuming and error-prone. Therefore, until software to facilitate the analysis of mobile eye tracking data in real world settings has been developed, the effort demanded needs to be weighed against the utility of the analysis. Additionally, we assert that ethical considerations must be included in the application of eye tracking. The eye tracking data should be used to help make surroundings safer or more comfortable for the tracked individual.

### Ethics and Conflict of Interest

The author(s) declare(s) that the contents of the article are in agreement with the ethics described in http://biblio.unibe.ch/portale/elibrary/BOP/jemr/ethics.html and that there is no conflict of interest regarding the publication of this paper.

### Acknowledgements

This research was supported in part by grant 1234-56 from the Swiss National Science Foundation.

We wish to thank Lykos Leukos of the United Animal Organisation for providing some ideas in this article.

## supplementary material


