# Supplementary material for: Mobile eye tracking applied as a tool for customer experience research in a crowded train station
Source: J Eye Mov Res. 2023 Jan 16;16(1):10.16910/jemr.16.1.1. doi: 10.16910/jemr.16.1.1 (PMC10624146; doi:10.16910/jemr.16.1.1)
Supplement: Supplementary file 1 [file jemr-16-01-a-SD1-01.pdf]

Appendix

Table 3. A description of all scenarios used for the study.

Scenario	Starting point	Aim	Scenario description
Catching a connecting train after crossing the underground passage	Platform 9/10 east (1)	Platform ¾ west (4)	“Imagine that you just arrived on platform 9/10. Now you have to catch your connecting train on platform ¾ west.”
Catching a connecting bus	Platform 9/10 east (1)	Tram/bus station (6)	“Imagine that you just arrived on platform 9/10. Now you have to catch your bus in front of the train station.”
Finding a luggage locker	Platform 9/10 east (1)	Storage area (5)	“Imagine that you just arrived on platform 9/10. Want to bring your luggage to the locker.”

Catching a connecting train on the same side of the passage	Platform 9/10 east (1)	Platform ¾ east (3)	"Imagine that you just arrived on platform 9/10. Now you have to catch your connecting train on platform ¾ east."
Catching a connecting train after crossing the underground passage – opposite direction	Platform ¾ west (4)	platform 9/10 east (1)	"Imagine that you just arrived on platform 3/4. Now you have to catch your connecting train on platform 9/10 east."
Catching a connecting train on the same side of the passage – opposite direction	Platform ¾ west (4)	platform 9/10 west (2)	"Imagine that you just arrived on platform 3/4. Now you have to catch your connecting train on platform 9/10 west."
Finding a point of interest	Platform ¾ west (4)	Exit to university (7)	"Imagine that you just arrived on platform 3/4. Now you have to go to the university."

Finding a specific location outside the train station– opposite direction	Train station platform ¾ east (3)	Train station exit toward the university (7)	“Imagine that you just arrived on platform ¾ east. You want to go to university.”
Entering the station from a specific location outside the train station	Train station exit toward the university (7)	Train station platform ¾ (3)	“Imagine that you just entered the train station coming from the university. You want to catch a train on platform 3/4.”
Passing through the train station only.	Tram/bus station (6)	Train station exit toward the university (7)	“Imagine that you just arrived with the bus at the train station. Now you want to go to university.”
From locker to specific location outside the train station	Storage area (5)	Train station exit toward the university (7)	“Imagine that you took your luggage out of the locker. Now you want to go to university.”
